# Robotic arm-assisted arthroplasty: The latest developments

**DOI:** 10.1016/j.cjtee.2021.09.001

**Published:** 2021-09-02

**Authors:** Xin Chen, Shu Deng, Mao-Lin Sun, Rui He

**Affiliations:** Center for Joint Surgery, Southwest Hospital, Third Military Medical University (Army Medical University), Chongqing, 400038, China

**Keywords:** Robotic arm, Arthroplasty, Hip, Knee, Clinical efficacy

## Abstract

Joint arthroplasty is an effective method for treating end-stage joint lesions and damages. Robotic arm-assisted arthroplasty, a rapidly developing technology that combines navigation technology, minimally invasive technology, and precise control technology of the robotic arm, can achieve accurate preoperative planning, optimal selection of implants, minimally invasive surgery, precise osteotomy, and accurate placement of the artificial joint. It has the characteristics of high accuracy and stability, and thus is more and more widely used in the field of joint surgery. In this paper, we systematically reviewed the application and clinical efficacy of robotic arm-assisted technology in hip and knee arthroplasty to provide reference for its future promotion.

## Introduction

Joint arthroplasty is an effective method for treating end-stage joint lesions and damages caused by various reasons as well as for rebuilding joint functions. The hip and knee joints which participate in most human activities and are more easily to be worn than other joints. Therefore, the most mature joint arthroplasty technology is widely used in parts. The precision and accuracy of hip and knee arthroplasty are particularly important. Though the surgical techniques and prosthesis design keep improving, traditional hip and knee arthroplasty are affected by the surgeons' technical levels, and the incidence of deviation in prosthesis position and alignment is still high. Insufficient precision and accuracy will have a negative impact on the long-term survival rate of the prosthesis.^1^

Computer-assisted orthopedic surgery can further improve the precision and accuracy of the arthroplasty as well as the long-term survival rate of the prosthesis. Computer-assisted orthopedic surgery is based on two technologies: navigation and robotic arm-assisted technology. Surgical planning can be completed in the navigation system before surgery, and the surgeons can receive feedback on the key nodes in real-time intraoperatively to correct the operation and improve the accuracy. However, since navigation can only give the surgeons feedback and warning, it cannot fundamentally solve the error caused by the operators' manual control during the operation, which is the main cause of surgical error, resulting in the fact that the navigation technology is not widely used in clinical practice.^2^ In order to overcome the inaccuracy of manual positioning during the surgery, robotic arm-assisted technology came into being. It utilizes a mechanized device (usually a robotic arm) that can interact with the environment and sensors, physically control the surgical instruments and prostheses according to the navigation signs and preoperative planning, and improve the precision of bone and protheses by accurately positioning the surgical instruments. It can also determine the next surgical step of the robotic arm according to the real-time data, which can overcome the errors caused by manual control and greatly improve the intelligence and accuracy of the surgery. In 1992, the robot technology entered the field of artificial joint surgery for the first time. It was the ROBODOC system (Integrated surgical systems, Davis, CA, USA), which had completed numerous total hip arthroplasties (THAs).^3^

As of now, there are three types of robotic arm-assisted systems, i.e., the active system, passive system and semi-active system.

With the active system, the surgeon does not need to directly manipulate the instruments, and the robot will independently perform the osteotomy. It includes three modules: computer-aided preoperative planning, robotic arm osteotomy and navigational positioning monitoring. When the robot executes the procedure, the surgeon can only stop the procedure by using the emergency shutdown switch and cannot modify the procedure. The representative product of this type is ROBODOC (Integrated surgical systems, Davis, CA, USA) and CASPAR (URS Ortho GmbH, Rastatt, Germany).

The passive system can provide three-dimensional (3D) simulation information based on the real world and provide quantitative feedback to monitor the progress of the operation. The robot system will not take any action independently, nor will limit the behavior of the surgeon. Part of the surgical steps will be performed under the continuous and direct control of the surgeon. The system monitors the progress of the operation and provides detailed information throughout the operation, e.g., the accuracy of osteotomy or reaming. Conventional instruments are still used during the operation, and recommendations given by the system can be rejected based on the judgment of the surgeon.

The semi-active system is a system that limits the range of movement of the surgical instruments controlled by the robotic arm through preoperative human-machine dialogue and realizes the amount of osteotomy planned before the operation. It is a tactile feedback system driven by the surgeon and is responsible for reaming and osteotomy. It can limit the amount of osteotomy through the tactile feedback system. This system includes three modules: computer-aided preoperative planning, robotic arm osteotomy, and navigational positioning monitoring. The surgeon can move the instruments freely within the pre-programmed boundaries, i.e., the virtual boundaries. All interactive actions are performed according to the preoperative planning, and the final manipulation and actions still depend on the surgeon. The representative product of this type is RIO (Robotic arm interactive orthopedic system, MAKO Surgical Corp., FL, USA) and ACROBOT (The Acrobat Co. Ltd., London, UK).^2^, ^3^, ^4^, ^5^

Among the three types, the semi-active system is currently the most mature and widely used robotic arm-assisted system in joint arthroplasty.

Robotic arm-assisted joint arthroplasty combines navigation, minimally invasive technology, and precise control technology of the robotic arm to achieve accurate preoperative planning, optimal selection of implants, minimally invasive surgery, precise osteotomy, and accurate placement of artificial joints. In this paper, databases of PubMed and Cochrane Library were searched from inception to May 2020 for relevant studies using the key words of “robotic-arm” “robotic-assisted” “computer-assisted” “computer navigation” “hip arthroplasty” “knee arthroplasty”.

## Robotic arm-assisted THA

### Development process of robotic arm-assisted THA

Active robots were used in the field of THA after they were introduced in the 1990s. The first generation of active robots gradually lost the market due to postoperative nerve injury, dislocation and other complications that eventually increased the risk of revision,^6^^,^^7^ as well as prolonging the operation time and increasing blood loss.^8^^,^^9^ Studies in different countries and regions have reported a reduction of 1%–22% in the application of active robot-assisted systems during the operation.^10^^,^^11^ Recently, with the launch of a new generation of active robot-assisted systems such as ROBODOC and MBARS, a series of new technologies such as non-positioning needle navigation and miniaturized robots have made robotic surgery more minimally invasive and easy to perform.^12^, ^13^, ^14^

The pre-operative assistant design platform of the new generation of robots can better assist the surgeon in formulating the position, anteversion angle, and abduction angle of the prosthesis and evaluating the condition of bone defect.^15^ RIO from MAKO Surgical Corp. is a semi-active robotic assistance system that combines computer navigation with a semi-active boundary-constrained robot reaming system to achieve accurate acetabular reaming and cup placement. It is easier for the surgeon to accept the semi-active system because he/she can control the robotic arm during the operation, which is more in line with the surgeon's operating habits. By combining human intelligence and accurate operation of the robot, the surgeon can focus on formulating the surgical plan and judging the efficacy of the operation, while the robot focuses on providing precise operations. The RIO system is currently the most widely used THA robot.

### Efficacy of robotic arm-assisted THA

#### Accurate positioning of the prosthesis

With reference to the concept of “improved safety zone” proposed by Callanan et al.^16^ in 2011, i.e., the abduction angle 30°–45°, anteversion angle 5°–25°, a large number of studies have confirmed that the position of the prosthesis after using the robotic arm is more precise. Domb et al.^17^ included 1980 THA patients who were divided into groups treated by traditional methods, robot-assisted system, and navigation system, respectively. They found that the robot-assisted system performed better than the other groups in placing the acetabular cup prosthesis into the “safety zone”, i.e., it could achieve more accurate positioning than traditional surgery and navigation-assisted surgery. Armirouche et al.^18^ performed a study on 103 cases of THA using the MAKO system, and the average anteversion angle of the acetabular cup prosthesis was (21.2 ± 2.0)° after surgery. Redmond et al.^19^ and Nawabi et al.^20^ confirmed that the robot-assisted system can significantly improve the accuracy of the placement of the acetabular cup prosthesis. The studies of Lim et al.^13^ and Hananouchi et al.^21^ also confirmed that the robot-assisted system could achieve accurate placement of the femoral prosthesis, reducing the varus and valgus of the femoral stem and better compression in the medullary cavity.

#### More suitable offset

El Bitar et al.^22^ reported that the offset of the robot-assisted THA was good after operation, and 91.8% of the offset was within 10 mm. It could avoid complications such as claudication and Trendelenburg gait, increased stress on the hip joint, and accelerated wear on the prosthesis. Chen et al.^23^ reported that MAKO system can help to guide femoral placement after registration and to restore the offset better than conventional THA, but the authors did not clarify whether the reaming was conducted by robotic arm or not.

#### Improved limb length discrepancy

The average lower limb length discrepancy (LLD) after conventional THA can reach 1–15.9 mm.^24^ When the affected limb is shortened by more than 10 mm or extended by more than 6 mm, the patient will obviously feel uncomfortable.^25^ With the help of the robot-assist system, the surgeon can successfully control the LLD within an acceptable range. El Bitar et al.^22^ reported that the LLD after THA using a robot-assisted system was within 10 mm, which was similar to the findings of Domb et al.^17^; but the results were not significantly different from the traditional surgical methods.

### Existing problems

#### No obvious difference in the short-term clinical efficacy

Although a few studies have reported that robot-assisted arthroplasty has the advantages of less intraoperative blood loss, low complication rate and short hospital stay,^25^^,^^26^ most of the existing studies on robot-assisted arthroplasty compared with traditional surgery have not found a significant difference in the short-term clinical efficacy. Nakamura et al.^27^ compared the clinical efficacy of the robot-assisted THA with traditional surgery through a 5-year follow-up and found that there was no significant difference between their Japanese orthopedic association scores. Other studies have also failed to find that robot-assisted systems had obvious advantages in the short-term clinical efficacy through the Harris hip score, Merle d'Aubigné hip score and other scoring systems.^23^^,^^28^ According to existing reports, it is not yet possible to prove that the robot-assisted arthroplasty has an advantage in the short-term clinical efficacy, and there is a lack of relevant literature on the long-term clinical efficacy of robot-assisted arthroplasty. Although functional scores did not differ significantly in the groups that underwent robotic-assisted and conventional THA, Han et al.^29^ founded that the scores in some medium-term studies were significantly higher after 2–3 years in patients who underwent robotic-assisted surgery, although the differences disappeared at 5 years.

#### High possibility of prolonged operation time

Most studies have confirmed that robot-assisted arthroplasty requires more time, which may be related to the need to insert a positioning needle and the existence of a learning curve. As the learning curve is overcome, the operation time will be shortened accordingly. Redmond et al.^30^ reported that the initial operation time of robot-assisted THA was (79.8 ± 27) min; after 70 operations were performed, the operation time was shortened to an average of (69.4 ± 16.3) min. The average operation time of traditional THA was 51 min. Domb et al.^31^ reported that the average time between robot-assisted THA and traditional THA was not statistically different, and the existing literature was not enough to prove that the operation time after using robotic arm-assisted technology will be shorter than the traditional surgery.

Robot-assisted systems have been used in THA for nearly 30 years. After decades of development, the existing robot-assisted systems have achieved great improvements in joint stability and reduction in dislocation rate/lower LLD/risk of revision. The core advantage of the robot-assisted system is that it can realize digital pre-operative design and accurate osteotomy according to the pre-operative plan. However, it is currently not applicable to revision cases.

Robot-assisted THA could obtain similar results as traditional methods with fewer intraoperative complications and more accurate positioning of the prosthesis. The disadvantage of the techniques is a longer surgery time, which may increase the risk of blood loss and infection. However, this shortcomings may be easily overcome considering the fast development of the robot technology, showing good potential for its further clinical application.

## Robotic arm-assisted knee arthroplasty

### Development process of robotic arm-assisted unicompartmental knee arthroplasty (UKA)/total knee arthroplasty (TKA)

The disadvantages of traditional knee arthroplasty include obvious early postoperative pain, significantly weakened strength of the quadriceps femoris muscle of the operated limb, greatly affected functional exercise, postoperative knee fibrosis, as well as short-term and long-term dysfunction. The minimally invasive technique represented by UKA can improve the above-mentioned deficiencies and defects. However, minimally invasive knee arthroplasty has higher technical requirements for surgeons, thus it is difficult to achieve a standardized and repeatable surgical effect.^32^

The technology of robot-assisted system is expected to overcome the uncertainty of knee arthroplasty in a minimally invasive environment by different surgeons and achieve consistent surgical results.

CASPAR and ROBODOC were the first active robots used in TKA. The first generation of joint surgery robots were active robots used in TKA, which lacked self-correction capability and was inefficient and difficult to apply during surgery. It also caused complications related to surgical instruments, and the surgical effect was no better than traditional methods. On this basis, semi-active robot was developed. In Europe, ACROBOT robots are widely used. In North America, the US Food and Drug Administration has approved MAKO and NAVIO surgical robots. As a new generation of robotic arms that are semi-active and with tactile perception, these robots have partially solved the defects of the previous generation, which enable the surgeon to actively control the robot and effectively avoid mistakes in complex lesion areas and take full advantage of the robot's fine cutting and accurate navigation. This semi-active system can avoid inaccuracy of the surgeon's osteotomy through the feedback mechanisms of visual feedback, tactile feedback, and auditory feedback, significantly improve the efficiency of the surgical robot, obtain a more precise alignment, and achieve the preoperative planning during the operation.

The proportion of robotic systems used in unicompartmental arthroplasty is increasing. Data in New York shows that the proportion of hospitals using robotic unicompartmental arthroplasty has increased from 15.3% to 27.4%, and the proportion of surgeons utilizing robot-assisted unicompartmental arthroplasty increased from 6.8% to 17.7% in 10 years.^33^

Chin et al.^34^ found that robot-assisted TKA and UKA led to better radiological outcomes, with no significant differences in the mid- and long-term functional outcomes compared with conventional methods by systematic review and meta-analysis on a total of 23 studies comprising 2765 knees. Kayani et al.^35^ prospectively assessed the early functional rehabilitation and hospital discharge time, and they found that robot-assisted operation speeded up the early functional recovery time and reduced length of hospital stay compared with conventional jig-based TKA.

### Efficacy of robotic arm-assisted knee arthroplasty

At present, there are no surgical robots that can be universally used on the market, all of which are closed systems. Robot from one company can only use knee prostheses from the same company. This means that we cannot use the same type of knee prosthesis to compare and evaluate different robot systems.

#### Clinical efficacy of robotic arm-assisted UKA

**Accurate positioning of the prosthesis** Clinical studies have confirmed that the accuracy of prosthesis position in UKA could be significantly improved after using the robotic arm-assisted system.^36^ Whether it is an image-dependent surgical robot based on preoperative CT scanning or a non-image-dependent robot, its role in improving the accuracy of prosthesis position in UKA is definite and repeatable. By comparing the efficacy of robotic surgery with traditional methods, Lonner et al.^37^ found that the accuracy of the tibial prosthesis in the coronal and sagittal planes was significantly improved: using traditional methods, the tibial prosthesis was prone to be placed in a varus position, with an average angle of 2.7°; while adopting robotic surgery, this average value could be reduced to 0.2°, and the tibial prosthesis is mostly in a neutral position. In their study, the authors analyzed the rotational positioning error of the prosthesis conducted by four surgeons who adopted robotic surgery and had relatively little surgical experience. The results showed that the rotational positioning error of the femoral prosthesis was only (1.04 ± 1.88)°, and the rotational positioning error of the tibial prosthesis was only (1.48 ± 1.98)°. This level of error was much lower than traditional surgical methods. Citak et al.^38^ conducted comparison of cadaver surgery and confirmed that the accuracy of the femoral prosthesis positioning increased by 3 times with the robot technology, while the accuracy of the tibial prosthesis positioning improved by 3.4 times. Bell et al.^39^ published the first clinical randomized controlled study in this field. By comparing the data of 120 patients (62 cases with MAKO robotic surgery and 58 with traditional surgery), the authors found that the robotic group was significantly higher in the proportion of tibial and femoral prosthesis positioning within 2° of the preoperative targets than the traditional group. As the accuracy of the prosthesis position increases, the accuracy of the ligament balance can also be improved. Pearle et al.^40^ found that in the evaluation of the comprehensive alignment of the lower limbs, the difference between the hip-knee-ankle angle after robotic arm-assisted UKA and the preoperative plan could be controlled within 1°–1.6°. The application of robotic arms to assist UKA can improve the balance of soft tissue, its accuracy can be controlled at 0.53 mm, and 83% of patients can be controlled within 1 mm under knee flexion. With this technology, the soft tissue balance is better, and the postoperative dynamics of the knee joint can be improved with better function and longer service life.

**Joint line height** Robotic arm-assisted UKA can better maintain the height of the joint line; the shallower the depth of tibial osteotomy, the more the tibia bone mass can be preserved. Ponzio et al.^41^ conducted a comparative study of robotic arm-assisted UKA *vs.* traditional surgery with the largest sample size to date, including 8421 cases of robot technology and 27,989 cases of traditional methods. It was found that a higher proportion of 8–9 mm polyethylene pads were used in the robotic group, which indirectly confirmed that the amount of tibial osteotomy was lower in the robotic surgery; meanwhile, in patients using more than 10 mm polyethylene pads, the robotic group accounted for only 6.4%, while the traditional group accounted for as high as 15.5%.

Reducing the amount of tibial osteotomy will benefit the patients in the following two aspects: (1) the more the tibial osteotomy, the lower the mechanical strength on the bone surface, the higher the probability of aseptic loosening of the prosthesis; (2) the more the tibial osteotomy conducted in the primary arthroplasty, the greater the probability of using wedge or extension stem to deal with the bone defect when receiving revision. Therefore, applying robot technology to reserve more bone mass for the tibia will effectively reduce the probability of revision and the difficulty of revision.

**Quality of life and mid-term survival rate** Judging from the early and mid-term results of the clinical studies, robotic arm-assisted UKA has obvious advantages over traditional surgery.^42^ Roche^43^ reported the 3-year follow-up results of 73 early cases, which showed an excellent postoperative knee joint function, with knee flexion reaching 129° in 2 years and 125° in 3 years. A multicenter clinical study found that the lowest 2.5-year prosthesis survival rate of 1007 consecutive cases was 96%, and 92% of the patients were satisfied or very satisfied with the surgery; randomized controlled trials revealed that the use of robotic systems was superior to traditional surgery for pain scores at 2 months and functional scores at 3 months postoperatively.^44^ Using the forgotten joint score system to evaluate patients' comprehensive joint function and proprioception, the number of patients who achieved 80% recovery in the robotic group was twice of that in the traditional group. At 1 year postoperatively, the scores of motor ability recovery in the robotic group were higher than that of the traditional group, such as the reduction of joint stiffness and the continuous improvement of the forgotten joint score and other functional scores. However, there is still a lack of long-term follow-up results. Although robotic surgery can significantly improve the accuracy of the prosthesis positioning, whether this improvement can bring about improved knee function and survival rate of the prosthesis remains to be confirmed by more clinical follow-up results.

#### Clinical efficacy of robotic arm-assisted TKA

Up to now, there are few clinical data on the use of robotic arm-assisted TKA. The mid-term follow-up results are mainly from the active robotic system ROBODOC. Clinical studies on the semi-active system RIO are scarce and are partly from *in vitro* experimental studies. No significant differences in range of motion were found between robot-assisted and conventional knee arthroplasty for TKA.^45^ Between-study heterogeneity was high for range of motion with no significant moderators identified.^46^

Kayani et al.^47^ respectively examined the total operating time and length of hospital stay. They reported that robot-assisted surgery significantly prolonged the surgical time, but shortened the early functional recovery time and reduced the length of hospital stay compared with conventional jig-based TKA.

**Accuracy of alignment** Generally, ±3° is accepted as the safety range for the alignment. Correspondingly, the deviation of alignment in robotic TKA can be controlled between 1° and 3° from the neutral position.^48^ Hampp et al.^49^ conducted 12 cadaver experiments using 6 robots and 6 traditional methods, compared the difference between postoperative prosthesis position and preoperative planning; and they found that using robotic surgery, the bone thickness of intraoperative osteotomy was closer to the preoperative plan and the postoperative alignment angle was more accurate. During the follow-up process, the authors conducted CT scanning and found that the postoperative prosthesis position were more consistent with the preoperative plan in the robotic group. Marchand et al.^50^ compared the alignments of 330 cases using robot technology before and after surgery. Patients who had alignment deformity no greater than 7° were completely corrected; 96% of whose alignment abnormalities were within valgus 3° and returned to normal after surgery.

Suero et al.^51^ compared 30 cases of robotic TKA with 64 cases of traditional TKA and found that the degree of variation in the robotic group was significantly lower than that in the traditional group, and the repeatability was significantly higher. Moon et al.^52^ reported the results of a postoperative alignment comparison study using the active robot ROBODOC. Compared with traditional instruments, there was no significant difference in the alignments of the lower limbs, but the accuracy of the femoral prosthesis rotation using the robot was significantly improved.^52^ Bellemans et al.^10^ reported the 5.5-year follow-up results of 25 cases receiving robotic TKA and found that the deviation of the prosthesis position in the three planes from the preoperative plan were within 1°; besides, the postoperative functional score was significantly improved compared with before surgery. Song et al.^53^ reported that 30 patients who had bilateral TKA during the same period, with one side receiving traditional surgery and the other robotic surgery. The results of 1-year follow-up showed no significant difference in functional score and knee mobility between the two groups, but radiographs showed the alignments in the coronal and sagittal planes of the robotic group were more precise. Four TKA studies^46^^,^^54^, ^55^, ^56^ involving 431 knees looked at component angle outliers, with meta-analysis showing that robot-assisted TKA produced significantly lower risk of misalignment in terms of coronal femoral component angle and sagittal femoral component angle. No significant differences in risk of misalignment were found in terms of coronal tibial component angle and sagittal tibial component angle when comparing robot-assisted TKA to the conventional methods.

**Soft tissue protection** From the conclusions of the current literature, we could see the incidence of surgical complications had no significant difference between the robotic surgery and traditional surgery. After retrospectively analyzing 70 cases of robotic surgery and 50 cases of traditional surgery, Siebert et al.^57^ proposed that robotic surgery reduced the incidence of postoperative soft tissue swelling and adverse events. Sultan et al.^58^ proposed that robotic TKA was superior to traditional surgery through literature review. The results and conclusion of the current literature at least indicate that robotic surgery is no less effective than traditional method in protecting soft tissues.

**Patient satisfaction** Marchand et al.^59^ used the Western Ontario and McMaster Universities Osteoarthritis Index to investigate the patients' satisfaction at 6 months after surgery. Among them, 20 cases received traditional surgery and 20 cases robotic surgery. They found that patients underwent robotic surgery had lower postoperative pain scores and higher satisfaction scores; besides, the robotic group also had a higher postoperative function score. A randomized controlled study by Liow et al.^60^ applied the SF36 quality of life scoring system to evaluate 31 cases of robotic surgery and 29 cases of traditional surgery; the results showed that the robotic group was significantly better than the traditional group in the quality of life scores. In an uncontrolled study, Kim et al.^61^ followed 32 cases of robotic TKA and found that the postoperative knee society score was significantly increased.

**Learning curve** Compared with the traditional TKA, the learning curve of robotic TKA is significantly shortened. Sodhi et al.^62^ analyzed 240 patients with robotic TKA performed by 2 experienced surgeons. Compared with the last 20 cases, the operation time of the first 20 cases was significantly longer. After proficient in robot technology, there is no significant difference in the operation time compared with traditional surgery. They concluded that after several months of familiarization and application, robot technology could reach a more efficient level. Fleischman et al.^63^ reported 2 surgeons used robotic TKA technology: 1 had post-fellowship for 2 years, and the other with 10 years of surgical experience. Each performed at least 48 cases of robotic surgery, and the learning curve existed in the first 10–15 cases. The overall patient adverse reactions did not increase. Siebert et al.^57^ found that the time of robotic surgery would be extended to 135 min initially, it fell to 90 min after the surgeons became proficient, and the preoperative planning time could also be reduced by an average of 15 min. The learning curve of robotic surgery is relatively short. On average, after about 15 surgeries, the surgeon can maintain a stable operation time that is comparable to traditional surgery.

### Existing problems

#### Prolonged operation time

For new technologies, many of them may face the problem of prolonging the operation time during promotion period. Surgeons often need a certain time to overcome the learning curve, thus, prolonged operation time at this stage is normal. Fortunately, the learning curve of robotic unicompartmental arthroplasty is relatively flat, so even young surgeons with little experience can complete this operation after training. A survey of 11 surgeons showed that UKA surgical robot requires an average of 8 operations to complete the learning curve; after these, the operation time was relatively stable without any abnormal increase.^64^

#### Complications of robotic surgery

Robotic surgery uses drills for osteotomy instead of sharp tools such as oscillating saw, and the incidence of damaging the lateral collateral ligament and cruciate ligament during osteotomy has been significantly reduced. Robotic UKA needs to place the optical target on the bone; meanwhile, to avoid intraoperative micro-movements, the fixation nail is usually 3 mm or more in diameter and adopts a thread design. Therefore, fixation holes are formed in the femur and tibia, and stress concentration will occur around the hole, thereby increasing the incidence of lower limb fractures. What's more, there may be infection and poor healing of the skin where the optical target is fixed.

Judging from the current studies, the advantages of the robotic group lie more in the accuracy of the alignment and the prosthesis positioning rather than the clinical scores. More prospective, randomized controlled studies with a larger sample size and longer follow-ups are needed to study its pros and cons. We believe that with the continuous development of artificial intelligence and robot technology, robotic arm-assisted arthroplasty will become a reliable technique for performing TKA.

## Conclusion

Robot technology can significantly improve the precision and accuracy of hip and knee arthroplasty. It allows surgeons to master the key skills at a faster speed and a more friendly learning curve. It also enables to have a more minimally invasive, more precise and accurate surgery. The postoperative effect is better, the patient's satisfaction is higher.

High medical cost has caused a huge economic burden on the government nowadays. Robotic surgery simplifies the surgical instruments and eliminates the need for more prosthesis models, which can improve the overall efficiency of the medical team and reduce surgical cost in the future. The application of robots in the medical field is bound to grow rapidly in the field of joint arthroplasty.

Robot information technology can help realize robot telemedicine. With the 5G mobile network, the greatly improved speed and the enhancement of computing capabilities have enabled remote network operation and high-definition video interworking without delay, thus realizing the real remote robotic joint replacement. China has a vast territory and a huge medical market, but the medical resources are unevenly distributed. Therefore, the remote robot technology for joint arthroplasty will surely have huge development potential in the future.

## Funding

This work was supported by the National Key R&D Program of China (No. 2017YFC0110705).

## Ethical statement

Not applicable.

## Declaration of competing interest

The authors declared that there were no conflicts of interest.

## Author contributions

Rui He conceived of the presented idea and supervised the study process. Xin Chen conducted the literature review and wrote the manuscript. Shu Deng and Mao-Lin Sun collected and screened the literature.
